# Prognostic and Clinicopathological Significance of SATB1 in Colorectal Cancer: A Meta-Analysis

**DOI:** 10.3389/fphys.2018.00535

**Published:** 2018-05-15

**Authors:** Jun Zhao, Yajun Tuo, Wei Luo, Shaojun He, Yifei Chen

**Affiliations:** ^1^Department of Respiratory and Critical Care Medicine, Qinghai Provincial People's Hospital, Xining, China; ^2^Department of Endocrinology, Qinghai Provincial People's Hospital, Xining, China; ^3^Department of Respiratory and Critical Care Medicine, Zhongnan Hospital of Wuhan University, Wuhan, China

**Keywords:** CRC, SATB1, overall survival, prognosis, meta-analysis

## Abstract

**Background:** A large number of studies have reported the aberrant expression of special AT-rich sequence binding protein 1 (SATB1) in colorectal cancer (CRC). However, the role of SATB1 in CRC is still controversial. Therefore, we performed this meta-analysis to elucidate the prognostic and clinical value of SATB1 in CRC patients.

**Methods:** We searched Web of Science, EMBASE and PubMed entirely in January 2018 to identify related articles. Pooled Hazard ratio (HR) was adopted to evaluate the prognostic value of SATB1 in CRC and odd ratio (OR) was used to assess the clinicopathological significance of SATB1 in CRC.

**Results:** Ten eligible studies containing 7 on prognosis and 9 on clinicopathological characteristics were finally included in the present meta-analysis. Results revealed that patients with high expression of SATB1 tended to have shorter overall survival (OS) (pooled HR: 1.64, 95% CI: 1.04–2.57). Besides, we also discovered that the expression of SATB1 was associated with histologic grade (OR = 1.88, 95% CI: 1.06–3.34), distant metastasis (OR = 1.43, 95% CI: 1.11–1.85) and lymph node metastasis (OR = 1.50, 95% CI: 1.03–2.19).

**Conclusion:** Broadly speaking, our meta-analysis demonstrated that high expression level of SATB1 was related to poor prognosis in CRC patients.

## Introduction

Colorectal cancer (CRC) is reported to be one of the primary health threats to mankind worldwide accounting for the second most common cancer in women and third in men (Siegel et al., 2014). Approximately 135,430 people were diagnosed with CRC and 50,260 deaths were associated with CRC in 2017 (Siegel et al., 2017). Over the past decade, huge efforts have been made by studies to identify new and powerful biomarkers that could early detect or accurately predict the prognosis of patients with CRC (Rodia et al., 2017). However, the 5-year survival rate of CRCs accompanied by metastatic complications is still under 10% (Wang et al., 2012). There is still a long way to go to evaluate the prognosis of CRC accurately.

The special AT-rich sequence binding protein 1 (SATB1) was initially reported by Kohwi-Shigematsu and his co-workers in 1992 which could selectively bind AT-rich DNA sequences (Dickinson et al., 1992). It is a thymocyte-specific matrix association region (MAR)-binding protein that connects specific DNA elements with its cage-like network (Kouzarides, 1999). It was suggested that SATB1, as a cell type-specific genome organizer, could regulate gene expression by simultaneously binding chromatin or chromatin-remodeling enzymes to the SATB1 network (Yasui et al., 2002). In recent years, numerous articles have revealed the significant role of SATB1 in the differentiation of early erythroid, development of T-cell and response to the stimulation of physiology (Alvarez et al., 2000; Wen et al., 2005; Cai et al., 2006). Apart from the discoveries of these physiological roles, considerable studies have been focused on SATB1, given its vital role in gastric cancer (Yuan et al., 2016), breast cancer (Patani et al., 2009; Kobierzycki et al., 2013; Zhang et al., 2015), and CRC (Brocato and Costa, 2015), which suggest its critical role in promoting tumor invasion and metastasis.

Based on the number of published papers, the role of SATB1 played in the initiation of CRC has been extensively studied. An increasing number of publications reveal that SATB1 expression in CRC is associated to the expression of β-catenin (Nodin et al., 2012), cyclin D1, MMP2, NF-kB, PCNA (Zhang et al., 2013) and S100A4 (Niu et al., 2015). Consequently, a great number of studies demonstrate that SATB1 is a biomarker that can predict poor progress for CRC patients (Brocato and Costa, 2015; Frömberg et al., 2018). In the present study, we aim to explore the association between the expression level of SATB1 and CRC clinical outcomes by quantitatively performing meta-analysis.

## Materials and methods

### Search strategy

We sought PubMed, Web of Science, and EMBASE in January 2018 using the following items: “SATB1,” “special AT-rich binding protein 1”; “colorectal or rectum or colon or rectal” and “cancer or tumor or carcinoma or neoplasm.” In addition, we also reviewed the possible associative references in the identified studies to totally obtain relevant articles.

### Inclusion and exclusion criteria

Articles were thought to be appropriate when they were in accordance with the following criteria: (1) cohort research on patients with CRC; (2) studies investigating survival outcome or the correlation between SATB1 expression and clinical variables, (3) articles presenting hazard ratios (HRs) or the message which we can manually calculate HR value and corresponding 95% CI; (4) articles written in English; (5) articles using immunohistochemical method to detect the expression level of SATB1. Articles were excluded when the following circumstances appeared: (1) duplicate publications; (2) studies that did not provide usable data; (3) experiments performed on animals. We included the latest published articles when studies carried out on the same population to avoid overestimating HR values.

### Extraction of data and quality assessment

All the eligible articles were reviewed by 2 investigators independently and any disagreement was solved by discussing with third person. Information as follows was gathered: first author's name, publication year, study origin, case numbers, status of distant metastasis, state of lymph node metastasis, status of TNM stage, histologic grade, measuring method, the value of HR and follow-up time. The basic information of the feasible studies were summarized in a format of table. Newcastle-Ottawa Scale score (NOS) is a well-known tool which comprises four key domains (quality of selection, comparability, exposure and outcomes) to evaluate the quality of study. Its highest score is 9 and the smallest is 0. When the score of a study was 7 or higher than 7, we thought the quality of the study was good and we assessed the quality of each included study according to NOS.

### Statistical analysis

Review Manager Version 5.3 Software was used to evaluate the prognostic and clinical value of SATB1 in CRC. Sensitivity analysis and publication bias were carried out by Stata software (version 12.0, Stata Corporation, USA). The association between SATB1 expression and OS in CRC was assessed by HR and the relation between SATB1 expression and clinical features were evaluated by ORs. We used Engauge Digitizer to obtain HRs values if HR values could not be obtained directly from the articles (Tierney et al., 2007). We employed fixed effect model in the absence of significant heterogeneity (*I*^2^ ≤ 50%), otherwise, we chose to perform random effect model (Dersimonian and Laird, 1986). The reliability of the total pooled results was tested using sensitivity analysis. Begg's test was applied to assess possible publication bias and no deviation was considered to be existed when *P* > 0.05.

## Results

### Study selection process

**Figure 1:**
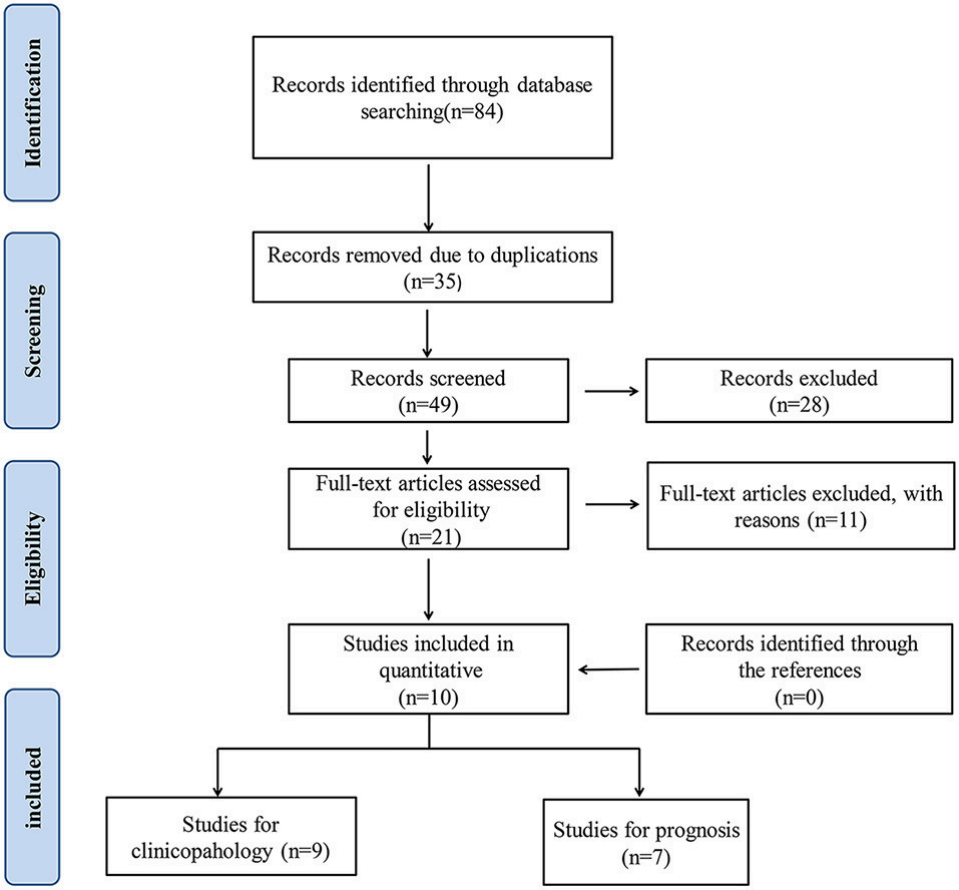
Flow diagram of study search and selection process.

As show in Figure 1, 84 articles were searched to be possibly eligible, among which, 35 were discarded due to duplicate publication. Then 49 articles were remained to be abstract scan. After we screened the titles and abstracts, 21 papers were full-text reviewed and 11 articles including 3 meta-analyses or reviews, 2 studies explored SATB1 expression in other kind of cancer, 6 without sufficient data were further discarded in accordance with the exclusion standards. Cross-referencing revealed no further relevant records, in the end, a total number of 10 articles including 9 studies for clinicopahology and 7 studies for prognosis were contained in our study.

### Characteristics of the included articles

Table 1 summarizes the detailed information on the basic characteristics of the included articles. The 10 studies comprise data from a number of 2456 CRC patients. Among these studies, six of them were carried out in China (Meng et al., 2012, 2015; Zhang et al., 2013, 2014; Niu et al., 2015; Lv et al., 2016) and one study each in Japan (Baba et al., 2016), Sweden (Nodin et al., 2012), Australia (Al-Sohaily et al., 2014), Poland (Kowalczyk et al., 2015). All the articles included in the present study measured SATB1 expression by immunohistochemistry. We obtained HR values directly from all the included studies except for Baba's (Baba et al., 2016) study.

**Table 1:** Basic characteristics of the included studies in this meta-analysis.

**References**	**Country**	**Total number**	**Histologic grade (W and M/P)**	**Distant metastasis(absent/present)**	**Lymph-node metastasis(absent/present)**	**TNM stage (I-II/III-IV)**	**Method**	**Follow up time (month)**	**HR**	**NOS score**
Baba et al., 2016	Japan	328	122/206	–	185/143	–	IHC	120	Survival curve	7
Nodin et al., 2012	Sweden	529	402/116	429/92	284/196	–	IHC	240	Reported	8
Al-Sohaily et al., 2014	Australia	341	7/323	–	–	166/172	IHC	120	Reported	7
Kowalczyk et al., 2015	Poland	102	–	87/15	55/47	51/51	IHC	60	Reported	7
Meng et al., 2015	China	132	–	–	–	–	IHC	204	Reported	8
Niu et al., 2015	China	131	23/108	111/20	–	59/72	IHC	80	Reported	8
Zhang et al., 2014	China	520	376/144	241/279	231/289	187/333	IHC	140	Reported	7
Zhang et al., 2013	China	80	54/26	–	–	32/48	IHC	–	–	7
Lv et al., 2016	China	200	42/158	189/11	99/101	97/103	IHC	–	–	7
Meng et al., 2012	China	93	12/81	–	46/47	24/69	IHC	–	–	8

*IHC, Immunohistochemistry; HR, hazard ratio; W, well; M, moderate; P, poorly*.

### Relationship between SATB1 and OS

**Figure 2:**
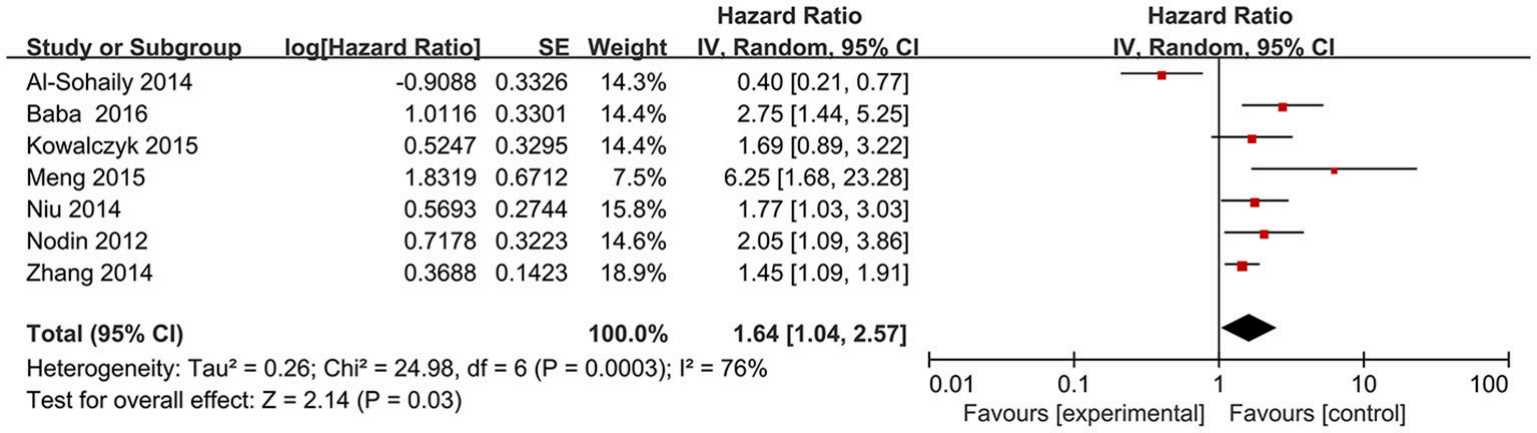
Forest plot of HR for the association between SATB1 expression and OS in CRC. OS, overall survival; CRC, colorectal cancer; HR, hazard ratios.

Seven studies (Nodin et al., 2012; Al-Sohaily et al., 2014; Zhang et al., 2014; Kowalczyk et al., 2015; Meng et al., 2015; Niu et al., 2015; Baba et al., 2016) explored the relation between the expression of SATB1 and OS in a number of 2083 patients. We applied random-effects model to explore their relationship since significant heterogeneity existed among articles (*I*^2^ = 76%, *P* = 0.0003). The data indicated that CRC patients with high SATB1 expression tended to have shorter OS (pooled HR: 1.64, 95% CI: 1.04–2.57, *P* < 0.001) (Figure 2).

### Relationship between SATB1 and clinicopathological characteristics

**Figure 3:**
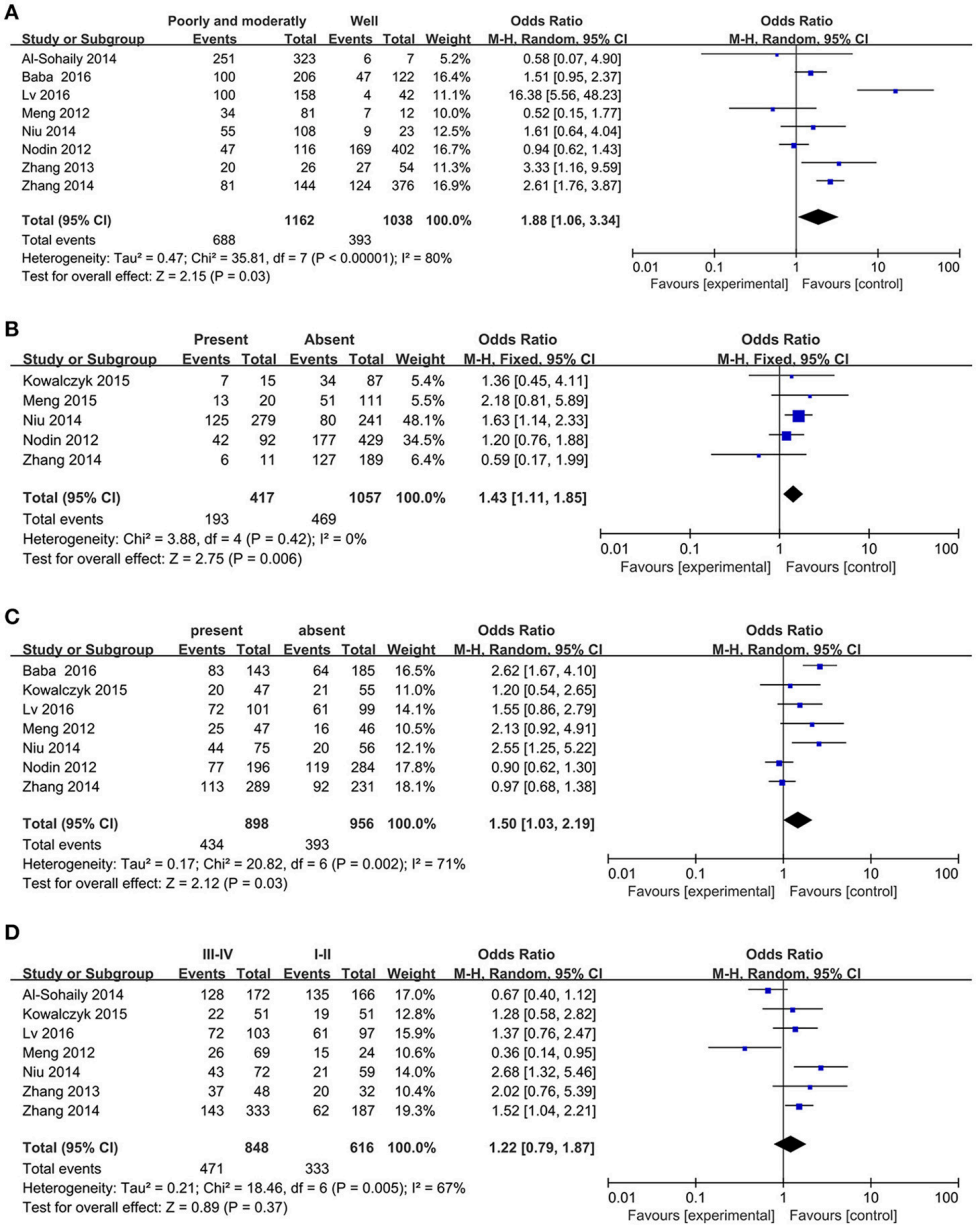
Forest plot for the relationship between SATB1 expression levels with clinical parameters in CRC. **(A)** Histologic grade; **(B)** Distant metastasis; **(C)** Lymph node metastasis; **(D)** TNM stage.

Eight studies provided the association between SATB1 expression and histologic grade status of the CRC patients with a number of 2222. Then we probed the relationship between them using random-effect model because of the significant heterogeneity (*I*^2^ = 80%, *P* < 0.01). The pooled OR was 1.88 (95% CI: 1.06–3.34, Figure 3A), which implied that high expressed SATB1 was associated with decreased tumor differentiation in CRC.

Five studies (Nodin et al., 2012; Zhang et al., 2014; Kowalczyk et al., 2015; Niu et al., 2015; Lv et al., 2016) containing 1482 patients explored the association between SATB1 and distant metastasis. Subsequently, we analyzed the association between SATB1 and distant metastasis. And the result elaborated that high level of SATB1 was associated with distant metastasis (OR = 1.43, 95% CI: 1.11–1.85, Figure 3B).

Among the included articles, seven of them (Meng et al., 2012; Nodin et al., 2012; Zhang et al., 2014; Kowalczyk et al., 2015; Baba et al., 2016; Lv et al., 2016)with a number of 1772 patients studied the relationship between SATB1 and lymph node metastasis. To probe whether a relation existed between SATB1 and lymph node metastasis, random-effect model was applied on account of the significant heterogeneity (*I*^2^ = 71%, *P* < 0.01). Results showed that patients with high expression of SATB1 tended to develop into lymph node metastasis (OR = 1.50, 95% CI: 1.03–2.19, Figure 3C).

Seven of the included articles shown the relation between SATB1 level and TNM stage of the CRC patients. We employed random-effect model to explore their association because the heterogeneity was significant (*I*^2^ = 67%, *P* < 0.01). It is a pity that the expression of SATB1 has no correlation with TNM stage (OR = 1.22, 95% CI: 0.79–1.87, Figure 3D).

### Sensitivity analysis and publication bias

**Figure 4:**
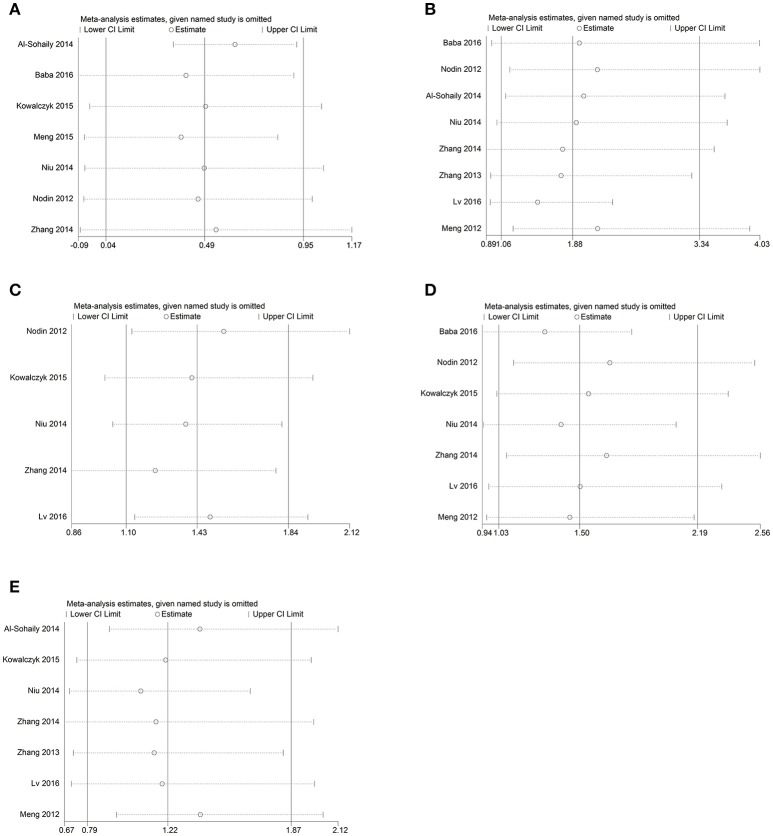
Sensitivity analysis of the studies. **(A)** overall survival; **(B)** Histologic grade; **(C)** Distant metastasis; **(D)** Lymph node metastasis; **(E)** TNM stage.

**Figure 5:**
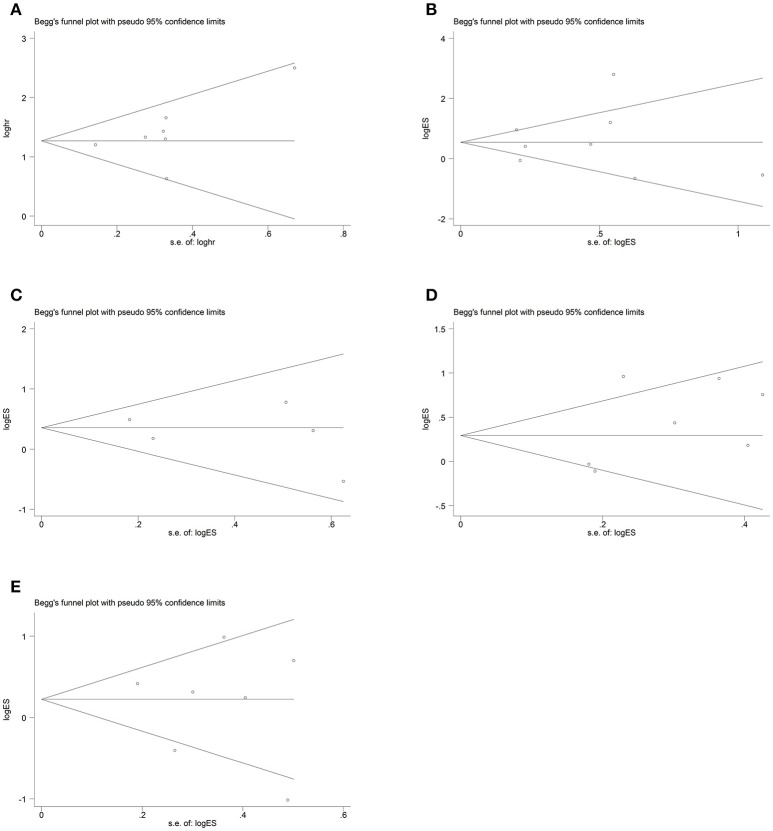
Begg's test for publication bias. **(A)** overall survival; **(B)** Histologic grade; **(C)** Distant metastasis; **(D)** Lymph node metastasis; **(E)** TNM stage.

To assess whether the individual studies affected the pooled HR or ORs, sensitivity analysis was performed. Articles were excluded one by one using stata software and none of them affected the result, conforming the stability of our data (Figure 4). Begg's test was adopted to assess publication bias and no publication bias was existed in our articles with all the *P-*values > 0.05(Figure 5).

## Discussion

CRC is a common tumor worldwide and more than 1 million newly diagnosed medical cases are identified every year (Patel and Ahnen, 2012). Although CRC can be cured at an early stage, its progression is rapid and the early clinical symptoms are hidden (Toiyama et al., 2014). Thus we should speed up to find novel biomarkers to diagnose CRC early. SATB1 is reported to be a tissue-specific MAR-binding protein which is involved in the packaging of chromatin structure (Zheng, 2008). The important role of SATB1 in cancers has been fully elaborated (Kohwi-Shigematsu et al., 2013). A great number of studies have reported the vital role of SATB1 in CRC (Meng et al., 2011; Frömberg et al., 2014; Mir et al., 2016). For example, Mir et al. (2016) showed that in CRC SATB1 expression was induced by hyper activation of Wnt/β-catenin signaling and repressed by depletion of TCF7L2 (TCF4) and β-catenin. Wang et al. found that decreased expression of PrPc can result in loss of SATB1 expression and reduced metastatic ability in CRC cells and the elevated expression of PrPc is associated with poor prognosis of CRC (Wang et al., 2012). In addition, the aberrant expression of SATB1 was reported to have a close relation to poor progress of CRC patients. All the above studies reveal the crucial role of of SATB1 in CRC.

Seven articles were included in the present meta-analysis for prognosis, 5 of which revealed that high SATB1 level was associated with poor prognosis. One showed no relation between SATB1 level and OS. Another paper even suggested that higher expression of SATB1 was associated with better OS. By pooling all the relevant articles, we concluded that high level of SATB1 was related to poor prognosis in CRC patients. The discrepancies maybe caused by the usage of different antibodies or the different expression between SATB1 mRNA and protein. We then explored the relation between SATB1 expression level and clinicopathological characteristics. The data revealed that high expression level of SATB1 was correlated with decreased tumor differentiation, distant metastasis and lymph node metastasis in CRC, indicating its important role in tumor progression and its possible use as a tumor marker. Up to date, many studies have explored the oncogenic mechanism of SATB1 in cancers, high expectations can be hold that SATB1 may be a novel tumor antigen (Frömberg et al., 2018).

A study performed by Zhang et al. which aimed to explore the prognostic value of SATB1 in gastrointestinal cancer also probed the role of SATB1 in CRC patients (Zhang et al., 2017). Comparing with their studies, our study should be more specific and reliable since we only included the data that detected SATB1 expression using immunohistochemical method. Besides more studies were included in our meta-analysis. Furthermore, in our study, we found that CRC patients with high SATB1 expression tended to have shorter OS (pooled HR: 1.64, 95% CI: 1.04–2.57, *P* < 0.001), however, in Zhang's study, although a trend of increased mortality for SATB1 overexpression in CRC patients with combined HR 1.55 was found, it was not statistically significant (95% CI: 0.97–2.49, *P* = 0.07). We think the discrepancies might be caused by the different inclusion criteria and number of included articles.

Among all the articles included in our meta-analysis, one article (Al-Sohaily et al., 2014) found that CRC patients with high expression of SATB1 tended to have high CpG island methylator phenotype (CIMP) status. Another article (Zhang et al., 2013) discovered that SATB1 overexpression was significantly correlated with BRAFV600E. It is well known that BRAFV600E plays pivotal role in CpG methlylation (Parsons et al., 2012). So we think that SATB1 may initiate CRC through affecting CpG methylation. However, more studies of high quality should be conducted to validate our hypothesis.

It should be noted that several limitations might exist in the present study. Firstly, different criteria of SATB1 positive expression were employed among the included studies, which may affect our conclusion to some extent; secondly, we obtained HR value of one study indirectly from Kaplane Meier survival curve, which might cause inaccurate results; thirdly, only manuscript in English were included, articles written in other languages might be missed; fourthly, most of the population of the studies are from China, so the result might tend to be more accurate in this population. Despite the limitations mentioned above, there are also some strengths of this study. All the studies included in this meta-analysis used immunohistochemistry to detect the expression of SATB1, which excluded the heterogenity caused by testing method. In addition, our article is a meta-analysis that combined results of multiple studies to probe the important role of SATB1 in the progress of CRC with strict inclusion and exclusion criterion.

Conclusively, our study firstly explored the relation between SATB1 and the clinical outcome of CRC patients. By combining all the data, we found that patients with high expression of SATB1 tended to have shorter OS and SATB1 was correlated with tumor differentiation, distant metastasis and lymph node metastasis. However, in view of the limitation of single study, more studies with larger population and high quality should be conducted to confirm our findings.

## Author contributions

JZ: Drafted the paper; YT and WL: Searched the databases and extracted the usable information; SH: Analyzed the data; YC: Designed the study and rephrased the paper. The paper was approved by all authors.

### Conflict of interest statement

The authors declare that the research was conducted in the absence of any commercial or financial relationships that could be construed as a potential conflict of interest.

The reviewer MV and handling Editor declared their shared affiliation.
